# Functional Characterization of Rare Variants in the *SHOX2* Gene Identified in Sinus Node Dysfunction and Atrial Fibrillation

**DOI:** 10.3389/fgene.2019.00648

**Published:** 2019-07-11

**Authors:** Sandra Hoffmann, Christoph Paone, Simon A. Sumer, Sabrina Diebold, Birgit Weiss, Ralph Roeth, Sebastian Clauss, Ina Klier, Stefan Kääb, Andreas Schulz, Philipp S. Wild, Adil Ghrib, Tanja Zeller, Renate B. Schnabel, Steffen Just, Gudrun A. Rappold

**Affiliations:** ^1^Department of Human Molecular Genetics, Institute of Human Genetics, University of Heidelberg, Heidelberg, Germany; ^2^DZHK (German Centre for Cardiovascular Research), Partner site Heidelberg/Mannheim, Heidelberg, Germany; ^3^Department of Internal Medicine II, University of Ulm, Ulm, Germany; ^4^Department of Medicine I, Klinikum Grosshadern, University of Munich (LMU), Munich, Germany; ^5^DZHK (German Centre for Cardiovascular Research), Partner site Munich, Munich, Germany; ^6^Preventive Cardiology and Preventive Medicine, Center for Cardiology, University Medical Center of the Johannes Gutenberg-University Mainz, Mainz, Germany; ^7^Department of General and Interventional Cardiology, University Heart Center Hamburg (UHZ), University Hospital Hamburg/Eppendorf, Hamburg, Germany; ^8^DZHK (German Centre for Cardiovascular Research), Partner site Hamburg/Kiel/Luebeck, Hamburg, Germany

**Keywords:** Shox2, transcription factor, cardiac conduction system, pacemaker, arrhythmias, atrial fibrillation, sinus node dysfunction

## Abstract

Sinus node dysfunction (SND) and atrial fibrillation (AF) often coexist; however, the molecular mechanisms linking both conditions remain elusive. Mutations in the homeobox-containing *SHOX2* gene have been recently associated with early-onset and familial AF. Shox2 is a key regulator of sinus node development, and its deficiency leads to bradycardia, as demonstrated in animal models. To provide an extended *SHOX2* gene analysis in patients with distinct arrhythmias, we investigated *SHOX2* as a susceptibility gene for SND and AF by screening 98 SND patients and 450 individuals with AF. The functional relevance of the novel mutations was investigated *in vivo* and *in vitro*, together with the previously reported p.H283Q variant. A heterozygous missense mutation (p.P33R) was identified in the SND cohort and four heterozygous variants (p.G77D, p.L129=, p.L130F, p.A293=) in the AF cohort. Overexpression of the pathogenic predicted mutations in zebrafish revealed pericardial edema for p.G77D and the positive control p.H283Q, whereas the p.P33R and p.A293= variants showed no effect. In addition, a dominant-negative effect with reduced heart rates was detected for p.G77D and p.H283Q. *In vitro* reporter assays demonstrated for both missense variants p.P33R and p.G77D significantly impaired transactivation activity, similar to the described p.H283Q variant. Also, a reduced *Bmp4* target gene expression was revealed in zebrafish hearts upon overexpression of the p.P33R mutant. This study associates additional rare variants in the *SHOX2* gene implicated in the susceptibility to distinct arrhythmias and allows frequency estimations in the AF cohort (3/990). We also demonstrate for the first time a genetic link between SND and AF involving *SHOX2*. Moreover, our data highlight the importance of functional investigations of rare variants.

## Introduction

Sinus node dysfunction (SND) and atrial fibrillation (AF) frequently coexist, both affecting the electrical activity of the heart. SND is a disorder of the dominant cardiac pacemaker (sinus node) caused by impaired automaticity and impulse transmission, which leads to abnormalities in heart rhythm such as sinus bradycardia, atrial tachyarrhythmias, alternating bradycardia and tachyarrhythmias, sinus pause, sinus arrest, and sinoatrial exit block (Choudhury et al., 2015). AF is the most prevalent cardiac arrhythmia, and genetic variation contributes substantially to AF susceptibility (Fatkin et al., 2017). The condition is characterized by episodes of uncoordinated electrical activity (fibrillation) in atrial cardiomyocytes, which cause a fast and irregular heartbeat.

SND affects up to one in five patients with AF (Jackson et al., 2017), while atrial arrhythmias are present in 40–70% of patients at the time of SND diagnosis (John and Kumar, 2016). Whether SND predisposes to AF or whether it manifests as a result of electrical remodeling induced by AF has been the subject of much debate (Kezerashvili et al., 2008). The molecular mechanism linking both conditions is still poorly understood. One hypothesis to account for the complex relationship is that the two conditions share a common genetic etiology.

Mutations in the homeodomain transcription factor gene *SHOX2*, a key regulator in cardiac development, especially the sinus node (Blaschke et al., 2007; Puskaric et al., 2010; Hoffmann et al., 2013), have been identified in both patients with early-onset and familial AF (Hoffmann et al., 2016; Li et al., 2018). Shox2 deficiency leads to severe bradycardia as demonstrated in different animal models (Blaschke et al., 2007; Espinoza-Lewis et al., 2009). Here, we performed a candidate gene study combined with functional analyses to identify a causal relationship between novel variants in the *SHOX2* gene and the development of SND and AF.

## Methods

### Study Participants

The SND patient cohort comprised 98 and the AF cohort 450 German individuals. SND cases were recruited from the Department of Medicine I of the Ludwig Maximilians University Hospital Grosshadern, Munich, from 2013 to 2014. Four hundred fifty participants from the Gutenberg Health Study (GHS) (Wild et al., 2012) enrolled in the period from 2007 to 2012 at the University Medical Center of the Johannes Gutenberg University Mainz, Germany, and were included in the AF cohort. Detailed patient characteristics are listed in **Supplementary Tables 1**, **2**, and **3**. Clinical parameters were available for at least 95% of all cases. The study was approved by the ethical commission of the Medical Faculty, University of Heidelberg, Heidelberg, Germany (S-104/2010 “Molekulare Grundlagen SHOX2-bedingter Herzfehlbildungen” 17.03.2010) and was performed in accordance with the ethical standards laid down in the 1964 Declaration of Helsinki and its later amendments. Every participant gave written informed consent including the consent to use their DNA for genetic analyses prior to the inclusion in the study.

### Mutational Screening

Mutational screening of the *SHOX2* gene was performed as described previously (Hoffmann et al., 2016). PCR primers used to amplify each exon of SHOX2 are listed in **Supplementary Table 4**. We analyzed the longest isoform of *SHOX2* including a primate-specific exon 2+ (NM_003030.4). PCRs were performed with Paq5000 polymerase (from Stratagene) using standard conditions. The PCR products were analyzed by gel electrophoresis. Adequate amplification products were selected for purification and sequencing done by Eurofins Genomics, Germany.

Data on identified variants have been uploaded to the gene variant database Leiden Open Variation Database https://databases.lovd.nl/shared/genes/SHOX2.

### Functional Analyses in Zebrafish

Human wildtype *SHOX2* complementary DNA was subcloned together with a *mlc2*-promotor into the pDestTol2CG2 vector that expresses green fluorescent protein under the control of the *mlc2*-promotor to generate an expression plasmid for injection in zebrafish embryos (TE4/6 wildtype strain), as described previously (Hoffmann et al., 2013; Hoffmann et al., 2016). The *SHOX2* mutations were introduced with the QuickChangeII Site-Directed Mutagenesis Kit according to the manufacturer’s instructions. All constructs were confirmed by sequencing. For overexpression experiments, 20 ng/µl of pDestTol2CG2-empty, pDestTol2CG2-SHOX2 wildtype, or pDestTol2CG2-SHOX2 mutant was microinjected into one-cell-stage embryos. Both morphological analysis and heart rate were determined after 72 hpf.

### nCounter Expression Analysis

Zebrafish hearts were isolated 72 hpf, and total RNA was extracted with the Direct-zol RNA Microprep Kit (Zymo Research) according to the manufacturer’s instructions. *Bmp4* levels were investigated by nCounter expression analysis at the nCounter Core Facility Heidelberg using the nCounter SPRINT Profiler. This RNA quantification technology allows target measurement from only 50 ng of input with high sensitivity and specificity. Twenty hearts per condition had to be pooled to obtain 50 ng of input material. In total, 40 hearts from two independent experiments were investigated per condition. Detailed probe design is given in **Supplementary Table 4**. The workflow is described at http://www.nanostring.com/elements/workflow. Background correction and normalization of data was performed using the nSolver Analysis Software 4.0 (NanoString Technologies). Most stable expressed genes were chosen for normalization based on the geNorm method.

### Cell Culture, Transfection, and Luciferase Assay

HEK293T cells were cultured at 37°C in Dulbecco’s modified Eagle medium containing high glucose, supplemented with 10% fetal calf serum and antibiotics. For luciferase assays, the published promoter of the SHOX2 target *BMP4* was used (Puskaric et al., 2010; Hoffmann et al., 2016). HEK293T cells were co-transfected with the respective pGL3-basic reporter construct (1µg) together with SHOX2 wild-type or mutant expression constructs (1 µg) using polyethylenimine (PEI). The *SHOX2* mutations were introduced with the QuickChangeII Site-Directed Mutagenesis Kit according to the manufacturer’s instructions. Twenty-four hours after transfection, luciferase activity was determined and normalized to Renilla luciferase activity with a dual luciferase assay kit (Promega). Experiments were performed independently four times (each sample measured in triplicate in each experiment) with consistent results.

### Statistical Analyses

GraphPad Prism7 (www.graphpad.com) was used to perform a one-way ANOVA combined with Tukey’s honestly significant difference *post hoc* test for multiple comparisons or followed by uncorrected Fisher’s least significant difference test. Differences in expression were tested by a ratio *t*-test.

## Results

### Molecular Findings

**Figure 1:**
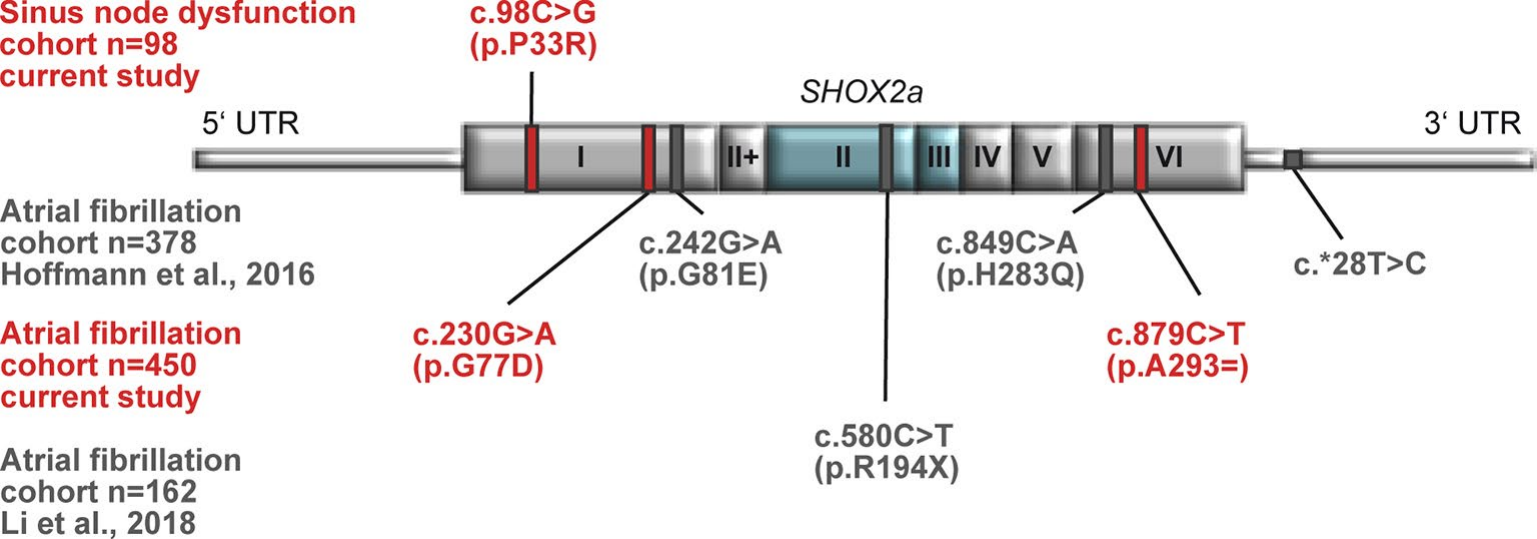
Identified SHOX2 variants in patients with sinus node dysfunction (SND) and atrial fibrillation (AF). Schematic drawing showing all identified variants within the *SHOX2* gene in patients with SND (upper part) and AF (lower part). The novel identified variants and the respective cohorts are highlighted in red. The published variants are depicted in gray. The homeobox that is encoded by exon 2 and 3 is highlighted in blue.

Sequencing of the coding exons of the longest *SHOX2* isoform was carried out in two study cohorts summarized in **Supplementary Tables 1** and **2**. A phenotypic relation between the study cohorts presents itself through the fact that 61.2% of all SND patients also suffer from AF. In total, we identified one heterozygous missense mutation (c.98C > G/p.P33R) in an individual suffering from SND (1/98) and four heterozygous non-synonymous and synonymous variants (c.230G > A/p.G77D, c.387G > A/p.L129=, c.388C > T/p.L130F, and c.879C > T/p.A293= ) in the AF (4/450) cohort (**Table 1**, **Supplementary Table 3**). Interestingly, the SND patient carrying the identified SHOX2 variant also presents with AF. *In silico* prediction based on “combined annotation dependent depletion” (C-scores) (Kircher et al., 2014) was used to predict the pathogenic potential of the identified variants. We selected all variants with C-scores ≥20 for further analyses, which included p.P33R (C-score = 25.8) of the SND cohort and p.G77D (C-score = 22.2) as well as p.A293= (C-score = 20.6) of the AF study cohort (**Figure 1** and **Table 1**). These variants are not present in the European Non-Finnish population of the 1,000 genomes project (Genomes Project et al., 2015). The genome Aggregation Database (gnomAD) (Lek et al., 2016) reports an allele frequency of 0.000009848% for p.P33R (1/50770) and 0.0004573% for p.A293= (55/60141) in the European Non-Finnish population, while the missense variant, p.G77D, identified in the AF cohort, was not reported.

**Table 1:** Overview of *SHOX2* variants identified in AF (atrial fibrillation) and SND (sinus node dysfunction) patients and control databases: 1,000 gnomes project (TGP) and the genome Aggregation Database (gnomAD); only the European Non-Finnish populations were considered. Novel variants were selected for further functional studies based on the predicted pathological potential *via* CADD (combined annotation dependent depletion).

Patient cohort	Genomic position (GRCh37)	Transcript consequence	Protein consequence	Patient cohort allele frequency	TGP allele frequency	gnomAD v2.1 (non-TOPMed) allele frequency	CADD score
Atrial fibrillation *n* = 378 Hoffmann et al., 2016	3:157823572 C > T	c.242G > A	p.G81E	0.0013227 (1/378)	–	0.00005404 (2/18504)	22.7
	3:157816035 G > T	c.849C > A	p.H283Q	0.0013227 (1/378)	–	–	25.2
Atrial fibrillation *n* = 162 Li et al., 2018	3:157820514 G > A	c.580C > T	p.R194X	0.0030864 (1/162)	–	–	38.0
Atrial fibrillation *n* = 450 current study	3:157823584 C > T	c.230G > A	p.G77D	0.0011111 (1/450)	–	–	22.2
	3:157822878 C > T	c.387G > A	p.L129=	0.0033333 (3/450)	–	0.0003306 (22/33275)	10.68
	3:157822877 G > A	c.388C > T	p.L130F	0.0011111 (1/450)	–	0.00001795 (1/27852)	9.02
	3:157816005 G > A	c.879C > T	p.A293=	0.0011111 (1/450)	–	0.0004573 (55/60141)	20.6
Sinus node dysfunction *n* = 98 current study	3:157823716 G > C	c.98C > G	p.P33R	0.0051020 (1/98)	–	0.000009848 (1/50770)	25.8

Together, these data expand the list of identified SHOX2 variants associated with AF and SND and confirm previous findings of SHOX2 defects in patients with early-onset and familial AF (Hoffmann et al., 2016; Li et al., 2018).

### Functional Analyses

**Figure 2:**
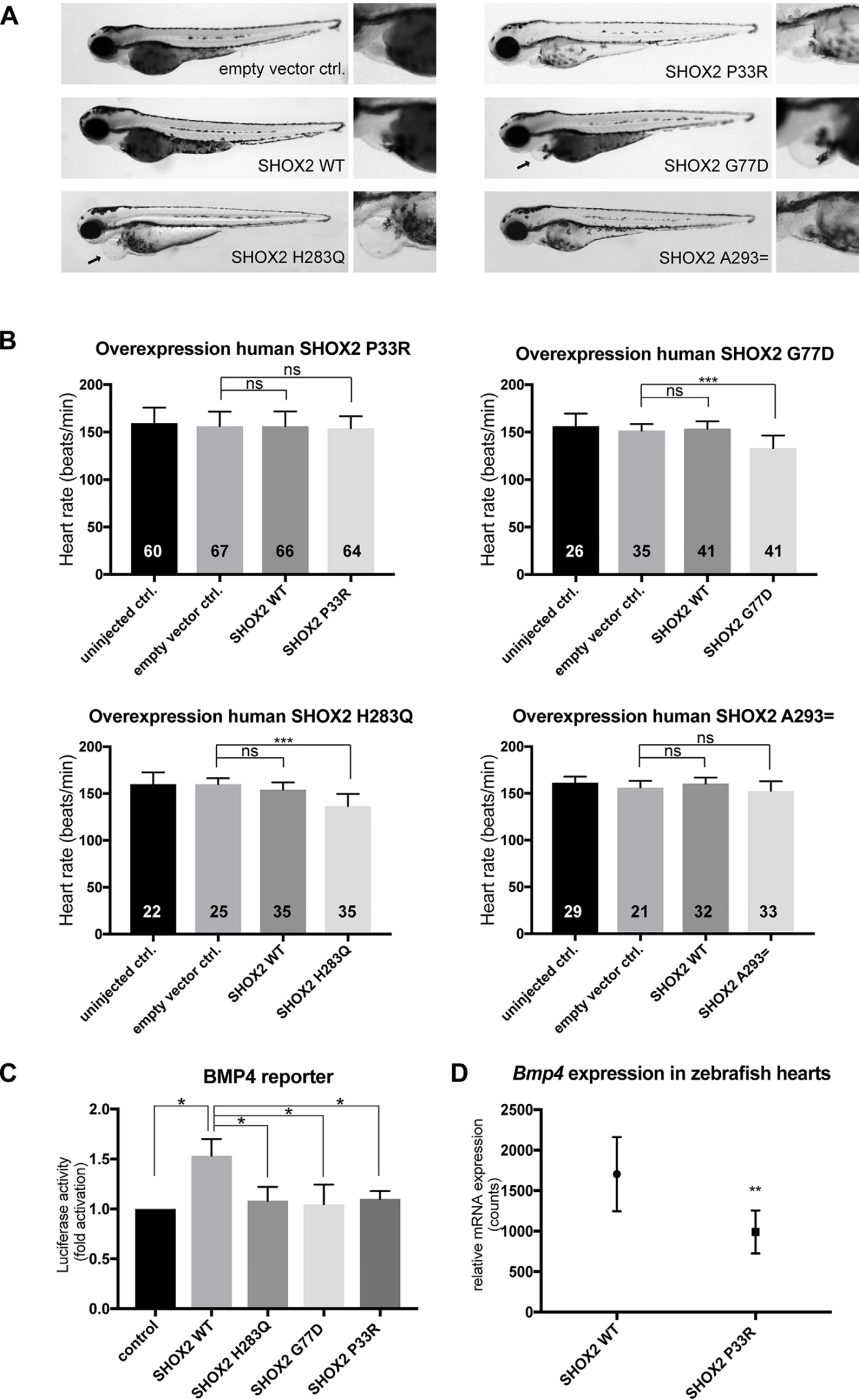
Functional characterization of SHOX2 variants *in vivo* and *in vitro*. **(A)** Cardiac-specific overexpression of SHOX2 mutants compared with SHOX2 WT (wild-type) leads to pericardial edema (arrow) for p.G77D and p.H283Q but not for p.P33R and p.A293= in zebrafish 72hpf. **(B)** The heart rate of zebrafish embryos was significantly reduced upon overexpression of p.G77D and p.H283Q but normal for p.P33R and p.A293= 72hpf compared with empty vector-injected embryos. SHOX2 WT overexpression showed no effect. Data are expressed as mean ± SD of three to six independent experiments. All *P*-values were determined by one-way ANOVA with Tukey’s multiple comparison test (*P < 0.05; ***P < 0.001; ns: not significant). **(C)** Luciferase activity of BMP4 reporter construct co-expressed with SHOX2 wild-type (WT) or SHOX2 mutants (p.H283Q, p.G77D, p.P33R) in HEK293T cells 24 h after transfection. All values are normalized to the empty pGL3 basic vector co-transfected with the respective expression constructs. Data are expressed as mean ± SEM of four independent experiments performed in triplicate. All *P*-values were determined by one-way ANOVA followed by uncorrected Fisher’s least significant difference test (*P < 0.05). **(D)** nCounter analysis revealed downregulation of *Bmp4* expression in zebrafish hearts (72 hpf) overexpressing the mutant p.P33R compared with wild-type SHOX2 (*n* = 40 hearts per condition from two independent experiments). Statistical differences were determined by a ratio paired *t*-test (**P < 0.01).

To investigate the functional relevance of the selected variants with regard to their pathogenic potential, we used zebrafish as a model system. In previous studies, we could demonstrate that the knockdown of Shox2 in zebrafish embryos leads to pericardial edema and severe bradycardia (Blaschke et al., 2007; Hoffmann et al., 2013). The previously identified missense mutation p.H283Q in patients with early-onset AF was investigated in this model, since it affects a highly conserved amino acid, also present in the zebrafish genome (zebrafish p.H277Q corresponds to human p.H283Q; **Supplementary Figure 1**) (Hoffmann et al., 2016). We could demonstrate that the zebrafish p.H277Q mutant severely affects the pacemaker function of Shox2, since it was not able to rescue the morpholino-mediated bradycardia phenotype (Hoffmann et al., 2016). In the current study, we used this mutant as a positive control and performed a cardiac-specific overexpression experiment (**Supplementary Figure 2**) of all identified human mutations compared with human wild-type SHOX2. By overexpression of the previously identified human p.H283Q variant, we observed a dominant-negative effect that resulted in pericardial edema and significantly reduced heart rates (**Figures 2A, B**). The missense variant identified in the AF cohort also revealed pericardial edema upon overexpression of p.G77D (**Figure 2A**). In addition, significantly reduced heart rates were observed for the missense variant p.G77D, while p.P33R and p.A293= showed no difference (**Figures 2A, B**). We also performed *in vitro* dual luciferase reporter assays to determine whether the identified missense variants have an impact on the transactivation activity of SHOX2. Wild-type SHOX2 activates the promoter of its target gene *BMP4* (Puskaric et al., 2010), while mutant SHOX2 harboring the previously reported p.H283Q variant severely affects the transactivation activity (Hoffmann et al., 2016). In the current study, we found that both novel identified missense variants p.P33R and p.G77D were unable to activate the *BMP4* promoter compared with wild-type SHOX2 (**Figure 2C**). This assay indicated for the first time that the variant identified in the SND cohort also leads to functional consequences. As the overexpression of the p.P33R mutant did not show phenotypic changes *in vivo* (**Figures 2A, B**), we examined whether it affected target gene expression. After cardiac-specific overexpression of p.P33R and wild-type SHOX2, the hearts of zebrafish embryos were isolated 72 hpf and subjected to comparative gene expression analysis using the nCounter technology. We could demonstrate a significant downregulation of *Bmp4* RNA levels upon overexpression of the p.P33R mutant (**Figure 2D**), which is in accordance with impaired Bmp4 reporter activation observed in the luciferase assay (**Figure 2C**).

The functional investigations in zebrafish combined with *in vitro* studies thus demonstrated a pathological potential for two of the three novel identified SHOX2 variants.

## Discussion

An orchestrated network of transcriptional regulators controls the proper function of the cardiac conduction system (van Weerd and Christoffels, 2016). Haploinsufficiency of a single cardiac transcription factor may contribute to cardiac conduction defects and arrhythmias (Seidman and Seidman, 2002; Mahida, 2014). We have identified the first heterozygous missense mutations (p.G81E, p.H283Q) in the *SHOX2* gene in a cohort of 378 patients with early-onset AF (Hoffmann et al., 2016). Recently, our findings were confirmed by the identification of a heterozygous nonsense mutation (p.R194X) in a patient with atrial fibrillation, which was absent in controls and co-segregated with the disease phenotype in a family with multiple affected members with complete penetrance (Li et al., 2018). Shox2 regulatory networks tightly control the pacemaker gene program in the developing sinus node and in nodal-like cells of the pulmonary vein myocardium, as demonstrated in different models (Puskaric et al., 2010; Hoffmann et al., 2013; Ye et al., 2015a; Ye et al., 2015b; Hoffmann et al., 2017). Several genes with pathogenic variants encoding specific ion channels (e.g. *SCN5A*, *HCN4*) and structural proteins (e.g. *ANK2*, *MYH6*) can result in phenotypes that manifest in both, SND and AF (John and Kumar, 2016). Here, we have extended the previous mutational analysis and also asked if *SHOX2* may serve as a common susceptibility gene for SND and AF to unravel a shared genetic etiology underlying both conditions.

A heterozygous missense mutation (p.P33R) was identified in the cohort of 98 SND patients. This variant was completely absent in the 1,000 genomes reference dataset and only reported in 1/50,770 European Non-Finnish individuals in the gnomAD database (Genomes Project et al., 2015; Lek et al., 2016). Notably, cardiovascular late-onset traits are not completely excluded in the gnomAD database, although participants from the National Heart, Lung, and Blood Institute Trans-Omics for Precision Medicine program (www.nhlbiwgs.org) involving heart, lung, blood, and sleep disorders were not included in the analyses. In a cohort of 450 AF patients, four heterozygous synonymous (p.L.129=; p.A293= ) and non-synonymous (p.G77D; p.L130F) variants were detected, which all reside outside the DNA-binding homeodomain. Combined annotation dependent depletion was used to further predict the pathogenic potential of the identified variants (Kircher et al., 2014). Any variant with a C-score ≥20 is considered to be within the top 1% of deleterious variants in the human genome. The C-scores of the identified variants range from 9.02 to 25.8. We selected all variants with C-scores ≥20 for further analyses, which included p.P33R (C-score= 25.8) of the SND cohort and p.G77D (C-score= 22.2) as well as p.A293 = (C-score= 20.6) of the AF study cohort. However, subsequent *in vivo* studies in the zebrafish model revealed a functional consequence only for p.G77D. Cardiac-specific overexpression of this missense mutation results in a dominant-negative effect with pericardial edema and significantly reduced heart rates. The synonymous variant (p.A293= ) with the lowest C-score (20.6) and highest allele frequency in the control population showed no difference upon overexpression compared to the wild-type. The missense variant p.P33R with the highest C-score (25.8) and comparatively low allele frequency in controls also showed no phenotypic effect in the in the zebrafish model, but molecular changes could be observed leading to an altered target gene expression of *Bmp4*. In addition, *in vitro* reporter assays demonstrated impaired transactivation activity for both missense variants p.P33R and p.G77D similar to the previously described p.H283Q variant.

A major challenge in clinical genetics is the interpretation of variants. Our study demonstrates that *in silico* prediction tools are not sufficient to determine the pathogenicity of a genomic variant; it highlights the importance of functional investigations *in vitro* and *in vivo* in model systems to distinguish pathogenic from nonpathogenic variants.

A comparison of all identified SHOX2 variants in AF patients (**Table 1** and **Supplementary Table 3**) shows remarkably that a strong functional significance with phenotypic expression could only be determined for those which have not been reported in public databases or were derived from individuals with an early disease onset before the age of 60 years (p.G77D, p.R194X, p.H283Q).

Although our data suggest a genetic link between SND and AF involving SHOX2, some study limitations have to be addressed. The small first SND cohort size (n = 98) may not be sufficient to detect rare SHOX2 variants with strong functional consequences. Assuming similar frequencies for rare pathogenic SHOX2 variants in SND and AF (3/990; 0.3%), additional studies are required to further delineate the role of SHOX2 deficiency in SND. Moreover, noncoding variants affecting regulatory elements were not addressed in the current study. Previously, a variant in the 3’UTR of the *SHOX2* gene has been described in patients with AF that mechanistically creates a functional microRNA-binding site (Hoffmann et al., 2016). Pathogenic *cis*-regulatory variants in enhancer regions can also not be ruled out.

Our findings strengthen the fact that rare heterozygous SHOX2 variants predispose to arrhythmogenic phenotypes including SND and AF. The novel identified and functionally characterized variants p.G77D and p.P33R confirm and expand previous findings regarding the association of SHOX2 with early-onset and familial AF.

## Contribution to the Field Statement

Cardiac arrhythmias contribute significantly to cardiovascular morbidity and mortality. Developing approaches toward disease prediction and treatment of heart rhythm disorders is therefore an important topic in healthy aging. Sinus node dysfunction (SND) and atrial fibrillation (AF) often coexist; however, the molecular mechanism linking both conditions is still poorly understood.

In the development of the cardiac pacemaker, the transcription factor Shox2 plays a pivotal role. Its deficiency leads to severe bradycardia in mouse and zebrafish. In humans, *SHOX2* mutations have been associated with familial and early-onset AF in a small patient cohort. Here, we asked if *SHOX2* may serve as a common susceptibility gene for SND and AF to unravel a shared genetic etiology. We extended our previous mutational analysis and identified two pathogenic predicted variants in 450 AF patients and one in 98 individuals suffering from SND. Investigations in zebrafish demonstrated functional consequences with phenotypic expression for one novel identified *SHOX2* variant in AF. In addition, *in vitro* analyses and molecular studies revealed an impaired biological function for two missense variants, identified in SND and AF. Thus, our findings suggest that rare *SHOX2* variants predispose to arrhythmogenic phenotypes including SND and AF and strengthen the hypothesis that the two conditions share a common genetic etiology.

## Data Availability

The datasets generated for this study can be found in LOVD, https://databases.lovd.nl/shared/genes/SHOX2.

## Ethics Statement

The study was approved by the ethical commission of the Medical Faculty, University of Heidelberg, Heidelberg, Germany (S-104/2010 “Molekulare Grundlagen SHOX2-bedingter Herzfehlbildungen” 17.03.2010) and was performed in accordance with the ethical standards laid down in the 1964 Declaration of Helsinki and its later amendments. Every participant gave written informed consent including the consent to use their DNA for genetic analyses prior to the inclusion in the study.

## Author Contributions

SH designed and performed the experiments, analyzed the data, and wrote the manuscript; CP, SAS, SD, BW, and RR performed experiments; SC, IK, SK, TZ, and RBS provided material or support; AS, PSW, AG, and SC analyzed the data; SJ and GAR designed the study, analyzed the data, and wrote the manuscript.

## Funding

This work was funded by the Deutsche Forschungsgemeinschaft (DFG) [RA 380/14-4] (GAR) and has received funding from the European Research Council (ERC) under the European Union's Horizon 2020 research and innovation programme (grant agreement No 648131), German Ministry of Research and Education (BMBF 01ZX1408A), and German Center for Cardiovascular Research (DZHK e.V.) (81Z1710103) (RBS).

## Conflict of Interest Statement

The authors declare that the research was conducted in the absence of any commercial or financial relationships that could be construed as a potential conflict of interest.
