# Supplementary material for: Functional Characterization of Rare Variants in the SHOX2 Gene Identified in Sinus Node Dysfunction and Atrial Fibrillation
Source: Front Genet. 2019 Jul 11;10:648. doi: 10.3389/fgene.2019.00648 (PMC6637028; doi:10.3389/fgene.2019.00648)
Supplement: Supplementary file 5 [file DataSheet_1.pdf]

```
                                                           p.P33R                                  p.G77D
Human        1    MEELTAFVSKSFDQKVKEKKEAITYREVLESGPLRGAKEPTGCT-EAGRDDRSSPAVRAAGGGGGGGGGGGGGGGGGG--  77
Chimpanzee   1    MEELTAFVSKSFDQKVKEKKEAITYREVLESGPLRGAKEPTGCT-EAGRDDRSSPAVRAAGGGGGGGGGGGGGGGGGG--  77
Pig          1    MEELTAFVSKSFDQKVKEKKEAITYREVLESGPLRGAKEPTGYA-EPGRDDRSSPAVRAAGGGGGGGGGGGGGGGGGGGG  79
Mouse        1    MEELTAFVSKSFDQKVKEKKEAITYREVLESGPLRGAKEP-GCV-EPGRDDRSSPAVRAAGGGGGAGGGGGG-GGGGGGG  77
Rat               --------------------------------------------------------------------------------
Chicken      1    MEELTAFVSKSFDPKAKEKKELITYREVLESGPLRGAREPGGAAAEPGRDETGSPA------------------------  56
Zebrafish    1    MEELTAFVSKSFDQKVKEKKEVITYREVLETGSVRN-RES--LSADPNREEISSIT------------------------  53
Frog         1    MEELTAFVSKSFDQKIKEKKEMITYREVLESGPARG-KEP-GCG-EGARED-------------GLAGNRCI-GGGGGG-  62

                     p.G81E
Human        78   VGGGGAGGGAGGGRSPVRELDMGAAERSREPGSPRLTEGRRKPTKAEVQATLLLPGEAFRFLVSPELKDRKEDAK--GME  155
Chimpanzee   78   VGGGGAGGGAGGGRSPVRELDMGAAERSREPGSPRLTEGRRKPTKAEVQATLLLPGEAFRFLVSPELKDRKEDAK--GME  155
Pig          80   AGGGGAGGGAGGGRSPVRELDMGAAERSREPGSPRLTE------------------------VSPELKDRKEDAK--GME  133
Mouse        78   AGGGGAGGGAGGGRSPVRELDMGAAERSREPGSPRLTE------------------------VSPELKDRKDDAK--GME  131
Rat          1    ---------------------MGAAERSREPGSPRLTE------------------------VSPELKDRKEDAK--GME  33
Chicken      57   -------GRAGGGRSPPREPDAAAADRAADAATPKLSD------------------------VSPELKERKEDAK--GME  103
Zebrafish    54   --------RSGVRSSPVREADMLASERSRDSSSPKLTD------------------------GNTDMKERKEDCK--PLE  99
Frog         63   ------GGGGGGARSPVLELDL-SVERIRESGSPKLTE------------------------VSPEIKERKEELKQQALE  111

                                                                 p.R194X
Human        156  DEGQTKIKQRRSRTNFTLEQLNELERLFDETHYPDAFMREELSQRLGLSEARVQVWFQNRRAKCRKQENQLHKGVLIGAA  235
Chimpanzee   156  DEGQTKIKQRRSRTNFTLEQLNELERLFDETHYPDAFMREELSQRLGLSEARVQVWFQNRRAKCRKQENQLHKGVLIGAA  235
Pig          134  DEGQTKIKQRRSRTNFTLEQLNELERLFDETHYPDAFMREELSQRLGLSEARVQVWFQNRRAKCRKQENQLHKGVLIGAA  213
Mouse        132  DEGQTKIKQRRSRTNFTLEQLNELERLFDETHYPDAFMREELSQRLGLSEARVQVWFQNRRAKCRKQENQLHKGVLIGAA  211
Rat          34   DEGQTKIKQRRSRTNFTLEQLNELERLFDETHYPDAFMREELSQRLGLSEARVQVWFQNRRAKCRKQENQLHKGVLIGAA  113
Chicken      104  DEGQTKIKQRRSRTNFTLEQLNELERLFDETHYPDAFMREELSQRLGLSEARVQVWFQNRRAKCRKQENQLHKGVLIGAA  183
Zebrafish    100  DETQTKIKQRRSRTNFTLEQLNELERLFDETHYPDAFMREELSQRLGLSEARVQVWFQNRRAKCRKQENQLHKGVLIGAA  179
Frog         112  EEGQTKIKQRRSRTNFTLEQLNELERLFDETHYPDAFMREELSQRLGLSEARVQVWFQNRRAKCRKQENQLHKGVLIGAG  191

                                                                      p.H283Q   p.A293=
Human        236  SQFEACRVAPYVNVGALRMPFQQDSHCNVTPLSFQVQAQLQLDSAVAHAHHHLHPHLAAHAPYMMFPAPPFGLPLATLAA  315
Chimpanzee   236  SQFEACRVAPYVNVGALRMPFQQDSHCNVTPLSFQVQAQLQLDSAVAHAHHHLHPHLAAHAPYMMFPAPPFGLPLATLAA  315
Pig          214  SQFEACRVAPYVNVGALRMPFQQDSHCNVTPLSFQVQAQLQLDSAVAHAHHHLHPHLAAHAPYMMFPAPPFGLPLATLAA  293
Mouse        212  SQFEACRVAPYVNVGALRMPFQQ------------VQAQLQLDSAVAHAHHHLHPHLAAHAPYMMFPAPPFGLPLATLAA  279
Rat          114  SQFEACRVAPYVNVGALRMPFQQDSHCNVTPLSFQVQAHVQLDSAVRAAHHHLHPHLAAHGPYMMFPAPPFGLPLATLAA  193
Chicken      184  SQFEACRVAPYVNVGALRMPFQQ------------VQAQLQLDSAVAHAHHHLHPHLA-HAPYMMFPAPPFGLPLATLA-  249
Zebrafish    180  SQFEACRVAPYVNVGALRMPFQQDSHCNVPPFSFQVQAQLQLDSAVAHAHHHLHSHLAAHAPYMMFPAPPFGLPLATLAA  259
Frog         192  SQFEACRVAPYVNVGALRMPFQQDSHCNVPSLSFQVQAQLQLDSAVAHAHHHLHPHLTAHAPYMMFPAPPFGLPLATLAA  271

Human        316  DSASAASVVAAAAAAKTTSKNSSIADLRLKAKKHAAALGL  355
Chimpanzee   316  DSASAASVVAAAAAAKTTSKNSSIADLRLKAKKHAAALGL  355
Pig          294  DSASAASVVAAAAAAKTTSKNSSIADLRLKAKKHAAALGL  333
Mouse        280  DSASAASVVAAAAAAKTTSKNSSIADLRLKAKKHAAALGL  319
Rat          194  DSASAASVVAAAAAAKTTSKNSSIADLRLKAKKHAAALGL  233
Chicken      250  ESASAASVVAAAAAAKTTSKNSSIADLRLKAKKHAAALGL  289
Zebrafish    260  ESASAASVVAAAAAAKTTNKNSSIADLRLKAKKHAAALGL  299
Frog         272  ETATAASVVAAAAAAKTSSKNSSIADLRLKAKKHAAALGL  311
```

Suppl. Fig. 1. Multiple sequence alignment of SHOX2 protein among different species. Novel identified variants are highlighted in red, while the previously reported variants are depicted in grey.

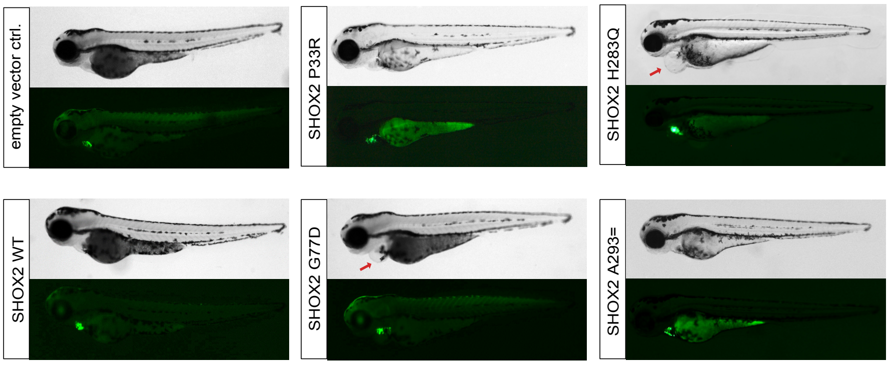

Suppl. Fig. 2. Fluorescence imaging of cardiac-specific overexpression of SHOX2 mutants in zebrafish. Bright field (upper image) and corresponding fluorescence imaging (lower image) of cardiac-specific overexpression of SHOX2 mutants compared to SHOX2 WT (wildtype) and empty vector control show pericardial edema (red arrow) for p.G77D and p.H283Q but not for p.P33R and p.A293= 72hpf.
